# Fever of Unknown Origin as a Sole Presentation of Subacute Thyroiditis in an Elderly Patient: A Case Report with Literature Review

**DOI:** 10.1155/2018/5041724

**Published:** 2018-10-25

**Authors:** Rishi Raj, Srujana Yada, Aasems Jacob, Dileep Unnikrishnan, Wael Ghali

**Affiliations:** ^1^University of Kentucky, Lexington, KY 40536, USA; ^2^Monmouth Medical Center, Long Branch, NJ 07740, USA

## Abstract

An 80-year-old Caucasian male presented with fever of 3-week duration. Outpatient workup for infectious etiologies was negative and due to persistent fever, he was hospitalized for further evaluation of fever of unknown origin (FUO). Physical examination and laboratory studies remained unremarkable; however a follow-up CT scan of chest, abdomen, and pelvis with contrast done to rule out malignancy as an underlying cause of FUO revealed heterogeneous thyroid gland with surrounding hazy changes suggestive of thyroiditis. Thyroid function tests confirmed the diagnosis of subacute thyroiditis. The patient was started on prednisone with good response in his symptoms and was eventually discharged to home. The importance of our case lies in the fact that diagnosing subacute thyroiditis in the absence of classical symptoms of neck pain can be challenging and a physician should have a very high index of suspicion especially in an elderly patient where FUO can be the sole presentation.

## 1. Introduction

Subacute thyroiditis (SAT) is the most common cause of painful thyroid swelling. It is supposed to be due to viral infection of thyroid gland and is relatively uncommon disease with incidence of 4.9 cases per 100,000/year with higher female to male preponderance [1]. Although, middle age female patients are most commonly affected, SAT has also been reported in extreme age groups [1–3]. Neck pain and tenderness is the most common presentation and is usually associated with signs and symptoms of hyperthyroidism [1–5]. While most patients with SAT can have constitutional symptoms, persistent fever in the absence of neck pain is uncommon. Diagnosis is mainly clinical which is confirmed with laboratory findings of thyrotoxicosis and radio iodine uptake scan showing reduced uptake [2]. Although not required for diagnosis, if done US thyroid gland shows enlarged and hypoechoic thyroid gland with irregular margin with presence of clustered epithelioid cells, scattered lymphocytes, and multinucleated giant cells on FNA [6]. NSAID and/or steroids are the mainstay of treatment [1, 2, 7]. Following resolution of transient thyrotoxicosis phase, about a quarter of patients develop permanent hypothyroidism requiring levothyroxine therapy [1–3].

## 2. Case Report

An 80-year-old Caucasian male presented to emergency room for evaluation of fever, headache, and recurrent falls for 3 weeks. Fever was intermittent, associated with chills at night and occasional occipital headaches, without any nausea, vomiting, visual changes, photophobia, phonophobia, or rash. He also had intermittent dizziness with multiple falls. There was no reported seizure activity or loss of consciousness. Review of symptoms was pertinent for intermittent chest pain, polydipsia, and polyuria but negative for palpitation, cough, shortness of breath, runny nose, ear pain dysuria, and weight loss. Patient had stable angina for which he was on as needed sublingual nitroglycerine; however for last three weeks he reported using the pills more frequently. There was no history of travel, tick bites, or sick contacts. His past medical history was significant for coronary artery disease, cerebrovascular accident with no residual deficit, corrected patent foramen ovale, diabetes mellitus type II, hypertension, hyperlipidemia, benign prostatic hyperplasia, and gout. His medications included aspirin 81mg daily, atorvastatin 80 mg daily, finasteride 5 mg daily, and tamsulosin 0.4 mg daily.

In the emergency room, on physical examination, he was febrile with a temperature of 101.4F, blood pressure of 162/80 mm Hg, pulse rate 110 beats/minute, respiratory rate of 18/minute, and saturating 98% on room air. Cardiovascular examination was significant for sinus tachycardia with a grade 2/6 systolic ejection murmur in aortic area. Respiratory examination revealed normal vesicular breath sounds in bilateral lung fields. Neurological examination was negative for any gross neurological focal deficits. There was no neck rigidity and Kernig's sign was negative. His abdominal examination revealed soft abdomen without any hepatosplenomegaly. Neck was supple without any thyromegaly or tenderness on palpation. There was no lymphadenopathy or skin rash. Rest of the physical examination was unremarkable.

**Figure 1:**
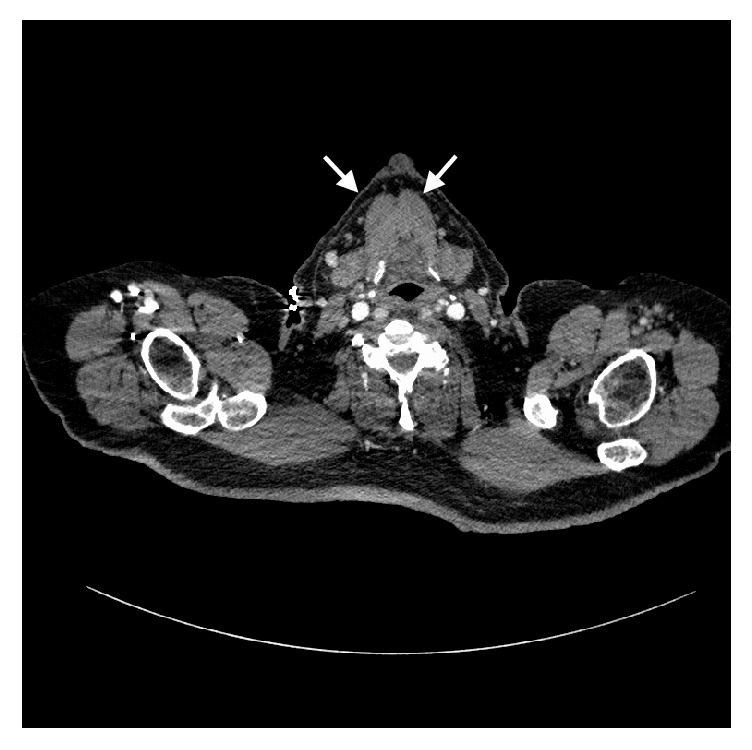
CT scan of neck with contrast showing mildly enlarged and heterogeneous thyroid glands (white arrows) (right>left) with surrounding hazy changes.

CT head was ordered due to the history of recurrent falls and was pertinent only for small old infarcts in left posterior pons. Patient requested to go home after initial laboratory investigation showed a normal white count (Table 1) and chest X-ray. Blood and urine cultures were drawn which remained negative. Patient returned after 4 days with no relief in his symptoms and was admitted for further evaluation. Inflammatory markers came back elevated with ESR of 86 mm/hr and CRP of 192 mg/L. Transthoracic and transesophageal echocardiogram were negative for any vegetations. As infectious etiology could not be found, undiagnosed malignancy was next among our differentials and therefore a CT chest, abdomen, and pelvis (Figure 1) with contrast were done which were unrevealing for any occult tumors but to our surprise showed a heterogeneous thyroid gland with surrounding hazy changes suspicious for subacute thyroiditis. Thyroid function tests were done as a follow-up which showed elevated Total T3 (6 ng/dL) and Free T4 (2.55 ng/dL) with low TSH (0.01 mIU/mL) suggestive of hyperthyroidism. Thyroglobulin (TG) antibodies and thyroid peroxidase (TPO) antibodies were undetectable. An ultrasound of the thyroid gland showed enlarged, heterogeneous thyroid gland involving right lobe (6.5 x 3.1 x 2.7 cm) and isthmus (1.4 cm in AP dimension), without any discrete nodules. Doppler studies revealed mildly increased intrinsic vascularity within the right thyroid. Retrospective review revealed TSH of 3.05 mIU/mL, 3 weeks prior to presentation.

Based on above findings, a diagnosis of subacute thyroiditis was made. As patient, received iodine contrast for CT scan of chest, abdomen, and pelvis with contrast, we could not perform radio-active iodine uptake (RAIU) studies. Patient was started on prednisone 40mg daily and dose of carvedilol was also increased from 3.125 mg to 12.5 mg to control cardiovascular symptoms of angina, which could have been related to cardiovascular effects of hyperthyroidism.

## 3. Discussion

Fever of unknown origin (FUO) was first described by Petersdorf and Beeson in 1961 as temperature > 38.3°C (>101°F) on several occasions, duration of fever for at least 3 weeks, and uncertain diagnosis after one week of inpatient investigations [16]. It is most commonly caused by infections, malignancies, rheumatologic, and inflammatory disorders; however in many patients the diagnosis remains uncertain even after extensive workup [17]. Although fever is often seen with many endocrine disorders, FUO as the sole presenting feature is very rare with only a few reported cases in the literature, majorly being from thyroid disorders [18, 19]. Our patient presented as a case of fever which did not reveal any diagnosis despite ambulatory workup and later found to be secondary to subacute thyroiditis (SAT).

Subacute thyroiditis (SAT) is a self-limiting condition, diagnosed in the presence of neck pain and/or tenderness, laboratory findings of hyperthyroidism (suppressed TSH and thyroid hormones), and elevated ESR with abnormal thyroid uptake [3]. Also termed as granulomatous, giant cell, or de Quervain thyroiditis, it accounts for about 5% of thyroid disorders and is thought to be due to viral infection of thyroid gland, often following an upper respiratory infections [20]. Most precise data on incidence and prevalence of subacute thyroiditis is from Olmsted County study by Fatourechi et al. which showed an incidence of 4.9 cases per 100,000/year [1960-1970] [1]. It is more common in middle age patients, most often between 40 to 50 years of age with a significantly higher incidence among females and is rare in elderly patients [1, 21]. Few studies have shown an increased incidence of SAT in summer (between July to October) which correlates with a viral infection to be involved in causality [2].

Typically, SAT presents with anterior neck pain and systemic symptoms of hyperthyroidism. Nishihara et al. did a retrospective review of 852 patients and found anterior neck pain to be the most common presenting symptom with the majority being unilateral neck pain (68.2%) followed by fever (28.2%) and symptoms of prior viral upper respiratory infection symptoms (23%) [2]. The neck pain is localized in the anterior aspect of the neck and can radiate to the jaw or ear. Neck tenderness and diffuse goiter are frequently present on examination. Low-grade fever, fatigue, weight loss, anorexia/increased appetite, tremor, and palpitation can also be seen during the thyrotoxic phase. Our patient did not have the classical clinical picture and was only noted to have tachycardia apart from persistent fever making the suspicion of SAT less likely.

Thyroid function tests follow a triphasic pattern. In the initial phase, transient hyperthyroidism occurs due to massive follicular destruction and is reflected on laboratory work up with suppressed TSH and elevated free T4 and Total T3. Following depletion of the preformed thyroid hormone, about 30% of patients go into a hypothyroid phase as reflected by elevated TSH and low thyroid hormones. This hypothyroid phase can last for several months before the thyroid function returns to euthyroid status [1–3]. Apart from abnormal thyroid function tests, inflammatory markers, e.g., erythrocyte sedimentation rate (ESR) and C- reactive protein (CRP), are usually elevated during the initial thyrotoxic phase [22, 23] and in our patient, both levels were noted to be elevated. Erdem et* al*. reported presence of antithyroglobulin antibodies in about 20 % of patients with SAT with a minor percentage of patients with positive Antithyroid peroxidase antibodies (4%) [15].

Although ultrasound of thyroid gland (US) is not necessary for diagnosis, when done in the initial stage it shows enlarged, hypoechoic thyroid gland with irregular margins. Gradually during recovery period, thyroid becomes normal; however in some cases, the hypoechoic areas continues to increases suggesting need for further medical treatment. Therefore, the main role of US in SAT is to assess disease evolution and to guide medical therapy and possibly detect early recurrence [6]. In a single-centric study, Solivetti* et al*. reported enlargement of juxtajugular and supra-isthmus lymph nodes on neck ultrasound in 91% of patients with thyroiditis and postulated it to be a sign for undiagnosed SAT. This study is promising, especially for diagnosing cases like ours where classical presentations were lacking [24].

Rarely, SAT presents with persistent fever as the sole clinical presentation and a careful review of literature showed almost all such cases had some associated symptoms as summarized in Table 2. Weiss* et al*. reported a case of an elderly male who developed FUO 2 weeks following coronary angiography and was found to have SAT which raises the possibility of iodine thyrotoxicosis; however in our patient there was no any precipitating event in prior 3 months [8].

Till date, there is no universal guideline for the management of subacute thyroiditis and is usually clinician driven [14]. The treatment is mainly symptomatic with analgesic and anti-inflammatory agents to control pain and inflammation and beta-blockers to control symptoms of thyrotoxicosis [7]. Usually, NSAIDs are used as the first-line agent to control the symptoms of pain; however in the presence of persistent symptoms despite use of NSAID or if there are severe symptoms, corticosteroids are used. Acute suppurative thyroiditis should be excluded prior to the start of steroids. While studies have shown shortened overall disease duration with use of corticosteroids in patients with SAT, higher incidence of hypothyroidism has been observed compared to those treated with only NSAIDs [1, 4]. However, it might also be due to sampling bias, with patients in steroid treatment group representing severe disease group with worse outcome. There is no established guideline regarding the dose or duration of prednisone therapy; however most of the existing literature suggest a starting dose of 40 mg daily and titrating down the lowest possible dose controlling the symptoms [1, 4, 12]. Low dose steroids (Prednisone 10 mg daily) have also been used in many reported cases with significant improvement in symptoms [11, 13, 14]. Our patient responded well to prednisolone 40 mg daily. Rarely, patient may need treatment with thyroidectomy for persistent neck pain despite treatment with steroids. Mazza et al. reported a case series of 3 patients who required thyroidectomy for persistent thyroid pain despite treatment with high dose steroid (prednisone 40 mg daily) for 2-3 weeks [25].

As about 20-30% cases can develop subclinical/overt hypothyroidism following resolution of transient thyrotoxicosis phase, a follow-up with thyroid function test is warranted [3, 19]. Among those patients, who develop hypothyroidism, there is a wide variation in terms of latency period. Fatourechi* et al*. described early (within 6 to 12 months) and late (after 1 year) hypothyroidism in 34% and 15% of the cases. This illustrates the need for both short and long term follow-up [1]. Once established, hypothyroidism should be treated with levothyroxine supplementation [1]. Recurrence of SAT is extremely uncommon although not unheard of and has been reported in about 1.6-4% of all the cases [1, 2].

In summary, presentation of subacute thyroiditis can be obscure among elderly patients due to several reasons. This can include factors such as lax skin overlying the thyroid gland masking a swollen thyroid gland, the presence of comorbidities muddling the subtle clinical findings, or inability of the patient to provide a good history. To avoid diagnostic confusion, it is important to keep SAT among the differential diagnosis of fever of unknown origin especially when an etiology cannot be established. We have summarized some of the recent literatures on SAT in Tables 2 and 3.

## 4. Conclusion

Painless thyroid gland with fever as the sole presentation is usually uncommon in SAT; however when there is no apparent cause for FUO, SAT should be considered especially in elderly patients.

## Figures and Tables

**Table 1:** Laboratory results.

Laboratory Test	Levels(Reference Range)

Hemoglobin	12.3g/dl (13-17.5g/dl)

White blood cells	10.1k/cmm (4.5-11k/cmm)

Neutrophils	78%(40-78%)

Lymphocytes	12% (20-45%)

Monocytes	9% (0-14%)

Eosinophils	1% (0-8%)

AST	44 U/L (8-48 U/L)

ALT	38 U/L (7-55 U/L)

Alkaline Phosphatase	99 U/L (45 to 115 U/L)

**ESR **	**86 mm/hr (0-20 mm/hr)**

**CRP **	**192.0 mg/L (<7.0 mg/L)**

HIV	Negative

Monospot test	Negative

IGRA	Negative

Hepatitis B surface antigen	Negative

Anti-hepatitis B core antibodies	Negative

Anti-hepatitis B surface antibodies	Negative

Anti-hepatitis C antibodies	Negative

Anti-hepatitis A antibodies, Total	Negative

Anti-hepatitis A antibodies, IgM	Negative

**Total thyroxine levels (Total T4) **	**13.4 mcg/dL (5.0-11.0 mcg/dL)**

**Free thyroxine levels (Free T4) **	**2.55 ng/dL (0.70-1.60 ng/dL)**

Total triiodothyronine levels (Total T3)	170 ng/dl (72-176 ng/dl)

**Free triiodothyronine levels (Free T3) **	**6 pg/ml (2.3-4.2pg/dl)**

**Thyroid stimulating hormone **	**0.01 mIU/mL (0.30-5.0 mIU/mL)**

Thyroid stimulating immunoglobulins	<89(<140)

Thyroglobulin (TG) antibodies	<1 IU/mL

Thyroid peroxidase (TPO) antibody	<1 IU/mL

**Table 2:** Summary of case reports with subacute thyroiditis (SAT) and fever of unknown origin (FUO).

Author/Year	Age/Sex	Summary
Weiss et al, 2000 [8]	81/M	(i) Fever, confusion, and bilateral lower extremity weakness. (ii) TSH 0.02 mIU/ml, FT4 3.1 ng/dL, FT3 6.0 pg/mL, and ESR 98 mm/hr (iii) Treated with Beta-blocker. (iv) Profound hypothyroidism at 3 months and euthyroid at 14 months.

Alexander et al, 2009 [9]	43/M	(i) Fever, weight loss and neck pain (ii) Treated with NSAIDs, beta-blockers and steroids (iii) Hypothyroidism on follow-up

Cunha et al, 2010 [10]	55/F	(i) Fever, chills, and night sweats, weight loss, fatigue, headaches, and jaw angle pain. (ii) Elevated ESR 98 mm/hr and CRP 84.5 mg/L (iii) Suspicion for Temporal arteritis but later found to have SAT. (iv) Elevated Ferritin levels 611 ng/mL (n = 10-291 ng/mL) (v) Suppressed TSH 0.009 mIU/mL, with elevated free T4 2.13 ng/dL and TBG antibody 332 IU/ mL (vi) FNA confirmed diagnosis of de Quervain's subacute thyroiditis

Karachalios et al, 2010 [11]	72/M	(i) Fever, neck pain, headache and weakness (ii) ESR 130 mm/hr, CRP 52 mg/dl (iii) Suppressed TSH 0.024 mIU/mL, elevated T4 185 nmol/l and normal T3 2.5 nmol/l (iv) TBG antibody and TPO antibody negative (v) Treated with Prednisone 10 mg daily for 2 weeks (vi) Asymptomatic at 2 weeks

Kim et al, 2013 [12]	48/F	(i) Fever, neck pain and history of URI 7 weeks prior (ii) Suppressed TSH 0. 065 *μ* IU/mL, elevated ESR 65 mm/h and CRP 2.07 mg/dL (iii) Treated with low-dose steroid (prednisone, 10 mg per day) (iv) Hypothyroid at 3 week with TSH 56.81 *μ*IU/mL and FT4 0.21 ng/dL.

Muqtadir et al, 2015 [13]	40/M	(i) Fever (ii) Elevated ESR 90 mm/h (iii) Suppressed TSH 0.02 mIU/mL and elevated FT4 3.66 ng/dL (iv) Negative TRAB and anti- TPO antibodies (v) US thyroid showed hypoechoic, heterogenous thyroid gland. (vi) Low radioactive iodine uptake (RAIU) (vii) Treated with Prednisone 10mg per day for 10 days. (viii) Hypothyroid at 3 week follow up.

Dalugama, 2018 [14]	42/M	(i) Fever, neck tenderness, cervical lymphadenopathy, anorexia, weight loss (ii) Raised ESR 80mm/hr and CRP 112 mg/L (iii) Suppressed TSH 0.012 mIU/mL with elevated free T4 42.08 pmol/L and Free T3 8.71 pmol/L (iv) FNA - clustered epithelioid cells, scattered lymphocytes, and multinucleated giant cells (v) Treated with Prednisone 10 mg daily for 7 days (vi) Euthyroid at 1 month.

**Table 3:** Summary of retrospective studies in various parts of world regarding epidemiology, clinical characteristics, treatment, and outcomes.

Author/ Year	Location	Results
Fatourechi Et al, 2003 [1]	Minnesota, USA (1960-1997)	N= 160 patients (F:M ratio 3:5), mean age 46 yr (range, 14–87 yr) Incidence 4.9 cases per 100,000/yr Clinical presentation (i) URI within 30 days in 20 (21%) and in 30-90 days in 4 (4.2%) patients respectively. (ii) Neck pain was most common symptom in 90/94 patients (96%) with radiation to jaw and ear in 12 and 18 patients respectively (iii) Less common symptoms - dysphagia in 30, myalgia in 12, tremor in 19, sweating in 22 & weight loss in 15 patients. Treatment (i) NSAIDs alone in 39 (41%) patients NSAIDs alone or NSAIDs + corticosteroids in 57 (61%) patients (ii) Corticosteroids in 34 (36%) patients. (iii) Thyroidectomy in 1 patient. Permanent hypothyroidism in 15% cases at end of 28 years. Recurrence rate 4% (with interval being from 6-21 years).

Benbassat et al, 2007 [4]	Israel (1999-2005)	N= 56 (Mean age 48.6+/-12 yr. Median disease duration 77 days. Treatment: (i) No treatment 5/ 56 patients. (ii) NSAID 43/56 patients. (iii) Steroids 25/56 patients. Follow up (i) Transient hypothyroidism 25/56 patients. (ii) Permanent hypothyroidism in 6/56 patients. Recurrence Rate 9%

Erdem et al, 2007 [15]	Turkey (1987-2001)	N= 176 (134 F and 35 M); mean age 34.0+/-17.8 yr Clinical presentation: (i) Thyroid pain in 97.1% of female patients & in 100% of male patients. (ii) Fever in 46% of patients. Laboratory studies: (i) Elevated ESR with mean ESR 43.42+/-39.68 mm/h. (ii) Anti-thyroglobulin antibody in 20%, & anti-TPO antibody in 4% of patients Recurrence rate: (i) NSAID group had recurrence rate of 10.6% in female patients and 12 % in male patients. (ii) Steroid group had recurrence rate of 17.5% in female and 10% in male patients.

Nishihara Et al, 2008 [2]	Tokyo, Japan (1996-2004)	N=852 patients (107 M & 745 F) with mean age 47.8±9.4 years (22-83 years). Presentation: (i) Unilateral neck pain in 68.2% of patients. (ii) Bilateral neck pain in 31.8% patients. (iii) Symptoms of URI in 23% of patients (iv) Fever in 28.2% Recurrence rate 1.6% with interval of recurrence 13.6±5.6 years.

Das, 2012 [5]	India (2010-2012)	N=12 patients (7 M and 5 F) with mean age 51 (range 45-60 years) Presentation: (i) FUO 10/12 patients (83.3%) (ii) Neck pain 11/12 patients (91.6%) (iii) Localized Erythema and pain 1(8.3%) (iv) Raised ESR and CRP in all (v) FNAC in 6/12 patients showed multinucleated giant cells. Treatment: (i) Analgesics (ii) Beta-blocker (iii) Steroids in 8/12 patients (66.6%) for severe symptoms. Recurrence in 2/12 (16.6%) Subclinical or overt hypothyroidism at 3 months in 7/12 patients (58.3%) patients.

Alfadda et al, 2014 [3]	Riyadh, Saudi Arabia (2004-2011)	N=25 (5 M and 20 F) with mean age 35.2 (range 3-78 years) Presentation: (i) Palpitation, painful goiter and weight changes were most common symptoms in 40-50% patients (ii) Anxiety, nervousness, fatigability and heat intolerance in about 20 % patients (iii) Change in appetite, tremors, fever, sweating, mood changes, insomnia and hoarseness of voice in < 20% patients Long Term Outcome: (i) Follow-up period 7± 4 years. (ii) Permanent hypothyroidism in 5 (20%) patients. (iii) Euthyroid state in 20 (80%) patients.
